# Gout Is Prevalent but Under-Registered Among Patients With Cardiovascular Events: A Field Study

**DOI:** 10.3389/fmed.2020.00560

**Published:** 2020-09-29

**Authors:** Irene Calabuig, Miguel Gómez-Garberí, Mariano Andrés

**Affiliations:** ^1^Sección de Reumatología, Hospital General Universitario de Alicante, Instituto de Investigación Sanitaria y Biomédica de Alicante (ISABIAL), Alicante, Spain; ^2^Departamento de Medicina Clínica, Universidad Miguel Hernández, Alicante, Spain

**Keywords:** gout, prevalence, cardiovascular event, cardiovascular disease, urate lowering therapy

## Abstract

**Objectives:** Gout is an independent cardiovascular (CV) risk factor with significant morbidity and mortality. We aimed to estimate the prevalence of gout, characteristics and management in a hospitalized population for CV disease, a topic that remains to be defined.

**Methods:** An observational, descriptive, cross-sectional study was carried out in patients admitted for CV events in the Cardiology, Neurology, and Vascular Surgery units of a tertiary center. Patients were selected following a non-consecutive, systematic sampling. Data about CV disease and gout were obtained from face-to-face interviews and patients' records. Gout diagnosis was established using the 2015 ACR/EULAR clinical classification criteria. The registration rate of gout was assessed by auditing patients' records and hospital discharge reports of CV events from the units of interest in the previous 2 years. To predict the presence of gout, multivariate logistic regression models were built to study the possible explanatory variables.

**Results:** Two hundred and sixty six participants were recruited, predominantly males (69.9%) and Caucasians (96.6%) with a mean age of 68 years. Gout was identified in 40 individuals; thus, the prevalence was 15.0% (95% CI 10.9–19.2%). In 35% of cases, the diagnosis was absent from patients' records. Gout was found in 1.4–2.6% of hospital discharge reports of CV events, also indicating under-registration. The disease was long-standing, but with low reported rates of flares, involved joints, and tophi. At admission, only half of the gout patients were on urate-lowering therapy, being 38.5% of them on serum urate <6 mg/dl. The only independent predictor of gout was the existence of previous hyperuricemia (median serum urate in previous 5 years ≥7 mg/dl), with an odds ratio of 2.9 (95% CI 1.2–7.1); if hyperuricemia is not included in the model, the only independent predictor was chronic kidney disease (odds ratio 3.0; 95% CI 1.4–6.6).

**Conclusion:** Gout is highly prevalent among patients admitted for CV events, with significant lack of awareness and suboptimal management, despite being a well-established independent CV risk factor.

## Introduction

Gout is a disease of monosodium urate (MSU) crystal deposition. It is the most frequent type of inflammatory arthritis in Western countries, with prevalence rates of up to 1% of the adult population and up to 5% of males aged 65 and over. Concerning Spain, a prevalence rate of 2.4% has been recently announced (1).

Many causes contribute to hyperuricemia, the key element in the pathophysiology of gout. One factor is urate overproduction, including inherited enzymatic defects and conditions with high cell turnover (psoriasis, hematological disorders) or dietary factors, such as elevated intake of fructose, proteins, purines, and alcohol. However, urate underexcretion, especially in the kidneys but also in the gut, constitutes the principal determinant of hyperuricemia (2). Also included are drug-induced hyperuricemia (especially by loop diuretics and thiazides), chronic kidney disease, hypertension, lead poisoning, and genetic disorders. In most cases, different genetic and environmental factors coexist and interact. Over recent years, urate transporter defects are gaining importance in the pathogenesis of hyperuricemia. Several genome-wide association studies have disclosed significant single-nucleotide polymorphisms (SNPs) in genes encoding these transporters, which result in a higher risk of hyperuricemia and gout (3). Interestingly, these studies often provide clues to uncover new urate transporters (4). Transporters URAT1 and NPT1 (present at proximal renal tubule) and also GLUT9 and ABCG2 (present at both proximal renal tubule and enterocytes) are the key regulators of serum urate (SU) levels in patients with hyperuricemia and gout (5). The rs2231142 variant of the ABCG2 gene and multiple variants of SLC2A9 (gene encoding GLUT9) are likely the most influent SNPs in SU concentrations (6). Besides, the ABCG2 rs2231142 allele is strongly associated with early-onset gout (7), with a sex-specific effect (as men present higher SU levels compared to women) (6, 8), and with a poor response to allopurinol (9).

MSU crystals form and deposit in tissues when SU levels remain above its saturation point (7 mg/dl)—the threshold for hyperuricemia (10). MSU crystal deposition depends on the level and duration of hyperuricemia, among other factors, such as advanced age, elevated body mass index, and male sex (11). When formed, MSU crystals are recognized by the innate immune system as danger signals, leading to recurrent episodes of acute arthritis. Between gout flares, a sustained low-grade inflammation persists (12). If not properly treated, clinically evident chronic inflammation with massive crystal deposits may take place. Therefore, gout is a systemic inflammatory disease beyond its well-known flares and is curable by proper treatment (13).

There is a firm association between gout and cardiovascular (CV) disease. Gouty patients develop atherosclerotic complications more frequently than the general population. This association was considered related to the traditional CV risk factors that are common in gout (14, 15). However, several recent studies have revealed that this association persists after adjusting for these factors, thus indicating that gout is an independent CV risk factor (16, 17). A 29% increase in mortality from any CV disease and a 42% increase in mortality from coronary heart disease is directly attributed to gout (18). Such increased CV risk is linked to systemic inflammation associated with MSU crystals, and to endothelial dysfunction and oxidative stress that occurs in hyperuricemia (19). Recently, potential deposition of MSU crystals at artery walls has been proposed (20, 21) but remains to be firmly established and deserves further research (22).

There is cumulative evidence that hyperuricemia has a pathogenic role in CV disease (23). Besides MSU crystals, soluble urate is also capable of unleashing an inflammatory response through innate immunity, which may intervene in the development of these diseases (24). Studies with animal models have described how hyperuricemia precedes and favors CV and renal diseases. Uric acid is able to stimulate NADPH oxidase, activate the renin-angiotensin system and impair nitric oxide release, which induces oxidative stress, endothelial dysfunction, renal vasoconstriction, and ischemia (25–27). A large amount of high quality evidence from epidemiological studies supports this hypothesis (28–31). Furthermore, urate-lowering agents have demonstrated CV and renal benefits (32, 33), especially in early-onset hypertension. Conversely, there are still Mendelian randomization, experimental, and epidemiological studies supporting the notion that hyperuricemia is not an independent CV risk factor (34, 35). At this point, we might recall the importance of the intracellular-extracellular uric acid dissociation. While extracellular uric acid takes part in the development of MSU crystal deposits (gout, kidney stones, and perhaps vascular calcification), intracellular uric acid is involved in the biological effects (hypertension and metabolic disease) (36). Also, no prospective study has assessed the occurrence of CV events on asymptomatic hyperuricemia according to the presence of subclinical MSU crystal deposits. These are estimated to be present in around 20% of subjects (37) and preliminarily linked to severe coronary atherosclerosis (38).

As with other chronic inflammatory diseases (39), early and proper management of gout likely allows control of the CV risk. When SU levels fall below the saturation point, MSU crystals dissolve, and gout manifestations subside. Consequently, the use of urate-lowering therapy (ULT) should also reduce the proatherogenic state in these patients. Available data are so far contradictory and derive from population-based studies. While some studies failed to demonstrate a CV benefit of ULT in gout (40, 41), Chen et al. (42) from Taiwan noted that the use of ULT significantly improved both CV and global survival rates. Besides, the failure to reach the SU level target (<6 mg/dl) (13) has been associated with poorer survival rates, mainly due to CV diseases (43). In addition to ULT, colchicine may also have a CV benefit. This agent, used in gout to prevent and treat flares, has been recently linked to lower rates of CV disease and mortality (44, 45). Also, there are positive data for secondary prevention of CV disease in the non-gouty population (46).

As the evidence supports the hypothesis that gout is an independent CV risk factor, with derived morbidity and mortality potentially avoidable, we consider it necessary and relevant to gauge the frequency and characterization of gout in patients admitted for CV events. The confirmation of high prevalence with suboptimal management would impact the interpretation and approach to both hyperuricemia-gout and CV disease. The CV risk control strategies initiated at the time of the event (secondary prevention)—usually focused on blood pressure, plasma lipids, and platelet activity—might benefit from proper gout management, based on the available evidence (42, 44).

The primary objective of this study was to estimate the prevalence of gout in patients admitted for CV events. The secondary objectives were (i) to assess the registration rate of gout in patients' records and discharge reports of CV events, (ii) to describe the characteristics and management of gout in this population, and (iii) to identify predictors for the presence of gout in patients with established CV disease. Our hypotheses were (a) that prevalence of gout in patients admitted for CV events would be markedly higher than in the general population, (b) that gout would be under-registered both in patients' records and discharge reports, and (c) that gout management in CV patients would remain suboptimal, with SU levels above the therapeutic target and insufficient use of ULT.

## Materials and Methods

We conducted an observational, descriptive, cross-sectional study in patients admitted for CV events in the Cardiology, Neurology, and Vascular Surgery units of our hospital, over 10 months (January to October 2018). It is an academic tertiary public hospital with a population coverage of 267,960 inhabitants (2017), eminently urban and with a slight female predominance (+4.4%). The coverage is broader, as Vascular Surgery and Neurology are reference units for other health departments, precisely in the care of atherosclerotic disease.

Adult patients hospitalized for a CV event were eligible. Events were defined by the clinical diagnosis registered in patients' records, as (i) acute coronary syndrome or coronary artery disease that requires revascularization, (ii) new or congestive heart failure, (iii) stroke or transient ischemic attack, or (iv) acute or chronic peripheral artery disease that requires revascularization. Patients were excluded if they presented a background of other inflammatory arthritis to avoid misclassification. Other exclusion criteria were declination or inability to sign the informed consent form. If excluded, minimal data (age, gender, CV event) were collected to ensure representativeness.

We followed a non-consecutive, systematic sampling. Screening for enrollment was applied to all patients admitted to the Cardiology unit the second week of odd months, the Neurology unit the fourth week of odd months, and to the Vascular Surgery unit the third week of even months.

Study variables were prospectively collected using a pre-established data collection form. Sources were primary care (SIA^®^) and specialized care (Orion Clinic^®^) electronic records and face-to-face interviews.

The primary outcome variable was the presence of gout, which was either previously registered (clinical or crystal-proven) at patients' records or defined by a face-to-face interview. For the interview, 2015 ACR/EULAR clinical classification criteria were used (47). Sensitivity of 85%, specificity of 78%, and area under the curve of 89% were published for the set clinical criteria (48).

To assess the registration rate of gout, we compared the face-to-face interviews with the patients' records. Likewise, we examined discharge reports by CV events from the units of interest in 2016 and 2017 with respect to the inclusion of gout as a secondary diagnosis (M10.X codes according to International Classification of Diseases, 10th edition); these data were provided by the Admissions and Clinical Documentation unit of our hospital.

Secondary outcomes and additional explanatory variables included SU levels at the time of the CV event (mg/dl); median SU levels in the previous 5 years (mg/dl); hyperuricemia (defined as a median SU level of ≥7 mg/dl in the previous 5 years or the level at admission if previous data were missing); being at the SU target (<6 mg/dl) (13); and others regarding the characteristics and treatments of gout. Other variables were demographic, clinical, and therapeutic related to CV disease (full list of variables available in the Supplementary Appendix). Chronic kidney disease (CKD) was defined as a median eGFR <60 ml/min/1.73 m^2^ in the previous 2 years or at admission if previous data were missing, estimated according to CKD-EPI formula (49).

The study complies with the Declaration of Helsinki and was evaluated and approved by the HGUA-ISABIAL Clinical Research and Ethics Committee [2018/07 act]. Informed consent was obtained from all subjects (or their legally authorized representative). This investigation is reported according to the criteria of the Strengthening the Reporting of Observational Studies in Epidemiology (STROBE) statement (50) and gout-related terms followed G-CAN nomenclature (51).

### Plan of Analysis

#### Sample Size

At the time of the study design, the prevalence of gout in Spain was undetermined. Later, a general population-based study communicated a prevalence of 2.4% (1). A former regional study carried out in Catalonia at the primary-care level estimated a gout prevalence of 3.3% (52), and this data point was used for sample size estimation. The present research focuses on population with CV disease, with a known increased risk of developing gout, so the prevalence was deemed to be close to 6%. On the basis of this estimation, a power of 80%, a statistical significance of 95%, and the assumption that 10% of patients would decline to participate in the study, a minimum sample size of 262 patients was calculated.

#### Statistical Analysis

Quantitative variables are shown as measures of central tendency (mean and median) with dispersion (standard deviations, interquartile ranges), and qualitative variables such as frequencies and percentages. Age was taken as continuous and categorized by tertiles.

For the primary outcome variable (prevalence of gout), 95% confidence intervals (95% CI) were calculated. For subgroup comparisons (clinical features according to gout diagnosis), Student's *t*-test, chi-square, and Fisher's exact test were used.

To assess which CV variables predict the presence of gout, a univariate analysis was initially performed for each explanatory variable with a chi-square test, and odds ratios were estimated by simple logistic regression. Those explanatory variables that were statistically significant for gout were included in a multivariate logistic regression model.

Statistical analyses were performed using IBM SPSS Statistics^®^ Version 25 (Armonk, NY). For the significance level, a *p*-value <0.05 was established.

## Results

Two hundred and ninety nine patients were screened for enrollment. After we excluded 33, the final study sample was 266 participants. The reasons for exclusion were a background of other types of arthritis (*n* = 16), inability to sign the consent (*n* = 9), and declination of participation (*n* = 8).

The general characteristics of the study participants are shown in Table 1. They were predominantly males, Caucasians, and elderly people, of advanced age. The CV events leading to admission were peripheral artery disease (47.4%; *n* = 126), stroke or transient ischemic attack (20.7%; *n* = 55), acute coronary syndrome (18.8%; *n* = 50), and heart failure (13.1%; *n* = 35).

**Table 1:** Characteristics of the study sample and comparison of clinical features regarding the diagnosis of gout.

	**Total (*n* = 266)**	**Gout diagnosis**		***p*-value**
		**No (*n* = 226)**	**Yes (*n* = 40)**	
Mean age (SD)	68.0 (12.0)	68.0 (13.0)	72.0 (9.0)	0.026
Mean BMI; (SD)	27.8 (5.1)	27.7 (5.1)	28.9 (5.1)	0.152
Males	186 (69.9)	154 (68.1)	32 (80.0)	0.132
Caucasians	257 (96.6)	217 (96.0)	40 (100)	0.363
Hyperuricemia (*n =* 252)	49 (19.4)	34 (15.8)	15 (40.5)	<0.001
Hypertension	201 (75.6)	168 (74.3)	33 (82.5)	0.268
Diabetes mellitus	143 (53.8)	122 (54.0)	21 (52.5)	0.862
Dyslipidemia	176 (66.2)	150 (66.4)	26 (65.0)	0.866
Tobacco consumption	70 (26.3)	61 (27.0)	9 (22.5)	0.552
Alcohol consumption	50 (18.8)	42 (18.6)	8 (20.0)	0.833
CV events leading to admission				
− Acute coronary syndrome	50 (18.8)	42 (18.6)	8 (20.0)	0.811
− Heart failure	35 (13.2)	29 (12.8)	6 (15.0)	
− Stroke	55 (20.7)	49 (21.7)	6 (16.0)	
− Peripheral artery disease	126 (47.4)	106 (46.9)	20 (50.0)	
Background of CV disease	147 (55.3)	122 (54.0)	25 (62.5)	0.318
Chronic kidney disease	74 (27.8)	52 (23.0)	22 (55.0)	<0.001
Use of diuretics	109 (41.0)	87 (38.5)	22 (55.0)	0.050

*Data shown as n (%), unless otherwise specified. BMI: body mass index; CV: cardiovascular; SD: standard deviation*.

Gout was identified in 40 individuals (prevalence: 15.0%; 95% CI 10.9–19.2%). Prior gout diagnosis in records was found in 26 patients (65.0%), either clinical-based (50.0%; *n* = 20) or crystal-proven (15.0%; *n* = 6). Thus, in the remaining 14 patients (35.0%), the diagnosis was unregistered but confirmed by interview. Rheumatology had previously seen all crystal-proven gout patients. Regarding discharge reports from the units of interest in 2016 and 2017 (*n* = 1,322 and 1,263, respectively), gout was coded as a secondary diagnosis in 19 (1.4%) and 33 (2.6%), respectively, also indicating under-registration.

**Figure 1:**
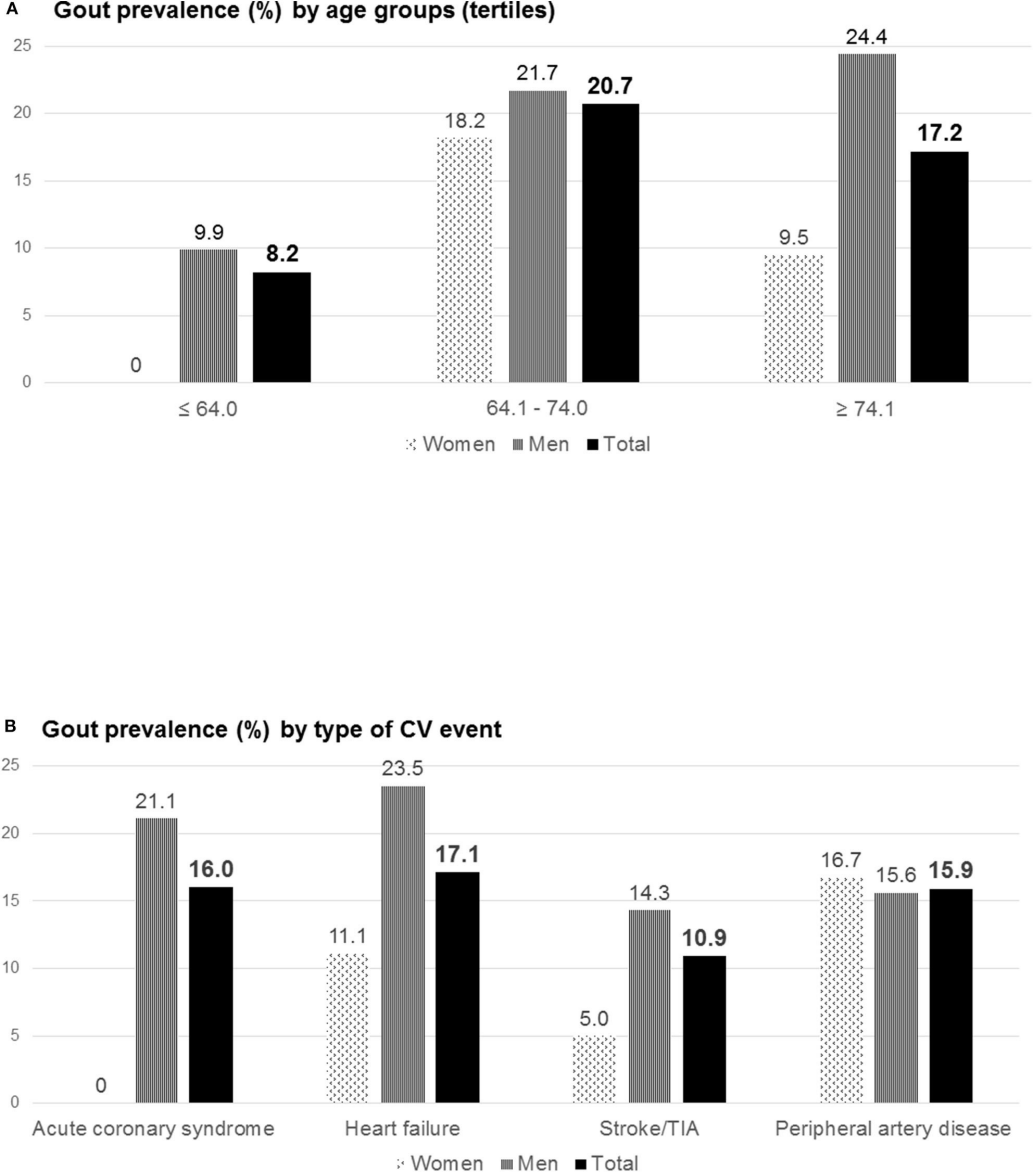
Gout prevalence (%) by age groups (tertiles) **(A)** and by type of CV event **(B)**. This figure shows gout prevalence in the whole population, separated by gender. The age groups were constituted according to the tertiles of the range of values in the distribution of age. The first group corresponds to patients aged 64 years or less, the second group ranges from 64.1 to 74 years, and the third group includes patients aged more than 74 years. CV, cardiovascular; TIA, transient ischemic attack.

The distribution of gout across age groups, gender, and type of CV event is shown in Figure 1. The prevalence of gout in those patients with established CV disease was 17.0%, while in those with a first event it was 12.6% (*p* = 0.318).

Table 1 shows the comparisons regarding the diagnosis of gout. Patients with gout were significantly older and showed higher rates of CKD and use of diuretics. No differences in other variables were observed.

The disease was long-standing though low numbers of flares and involved joints were referred (Table 2). Tophi were not seen in many patients. SU levels were not adequately controlled, either at the time of the CV event or after analyzing the previous 5 years. Despite 70% of patients' having received ULT at some point, only half remained treated at admission. Only one-third of the patients were at the SU target at admission (under ULT, 38.5%). Use of prophylactic colchicine was scarce, in contrast to the ample use of NSAIDs despite the CV context.

**Table 2:** Clinical features and management of the 40 identified patients with gout.

	**Gout (*n* = 40)**
Years since the first flare, median (IQR)	15 (10–30) (*n* = 39)
Number of flares, median (IQR)	3 (1–9) (*n* = 39)
Number of involved joints, median (IQR)	2 (1–3) (*n* = 39)
Presence of tophi	3/39 (7.7)
Serum urate levels (mg/dl), mean (SD) - at admission - median levels of the previous 5 years	7.1 (2.6) (*n* = 24) 6.8 (1.6) (*n* = 33)
Serum urate <6 mg/dl - at admission - median levels of the previous 5 years	8/24 (33.3) 7/33 (21.2)
Urate-lowering agents (ever)	28/40 (70.0)
Urate-lowering agents (current)	20/40 (50.0)
Allopurinol - current use - dose (mg/day), mean (SD) Febuxostat - current use - Febuxostat dose (mg/day), mean (SD)	16/20 (80.0) 200 (102) 4/20 (20.0) 80 (0)
Prophylactic colchicine (ever)	11/40 (27.5)
Prophylactic colchicine (current)	5/40 (12.5)
Use of NSAIDs for gout flares	25/40 (62.5)

*Data shown as n (%) unless otherwise specified. Number in brackets accounts for complete data. IQR, interquartile range; NSAIDs, non-steroidal anti-inflammatory drugs; SD, standard deviation*.

Simple and multivariate logistic regression models are shown in Table 3. Hyperuricemia was the only independent predictor of gout (OR 2.9; 95%CI 1.2–7.1). After having excluded hyperuricemia from the model, the only variable significantly associated with the presence of gout was CKD (OR 3.0; 95% CI 1.4–6.6).

**Table 3:** Clinical predictors for the presence of gout in the study sample.

	**Univariate**		**Multivariate (with hyperuricemia)**		**Multivariate (without hyperuricemia)**	
	**OR (95% CI)**	***p*-value**	**OR (95% CI)**	***p*-value**	**OR (95%CI)**	**p-value**
Sex (male)	1.87 (0.82–4.26)	0.136				
Age^*^	1.03 (1.00–1.06)	0.065				
Age >65 years	2.60 (1.15–5.90)	0.022	2.04 (0.78–5.34)	0.147	1.59 (0.66–3.87)	0.305
BMI^*^	1.05 (0.98–1.11)	0.153				
Obesity	1.93 (0.96–3.89)	0.065	1.56 (0.70–3.47)	0.275	1.66 (0.78–3.54)	0.190
Hypertension	1.63 (0.68–3.88)	0.272				
Diabetes	0.94 (0.48–1.85)	0.862				
Dyslipidemia	0.94 (0.47–1.91)	0.866				
Median uricemia 5 PY^*^ (*n =* 219)	1.94 (1.45–2.60)	<0.001				
Hyperuricemia (*n* = 252)	3.63 (1.71–7.70)	<0.001	2.64 (1.12–6.19)	0.026		
Tobacco	0.79 (0.35–1.74)	0.553				
Alcohol	1.10 (0.47–2.55)	0.833				
Current CV event - ACS - CHF - Stroke or TIA - PAD	1.00 (ref) 1.09 (0.34–3.46) 0.64 (0.21–2.00) 0.99 (0.41–2.42)	0.814 0.889 0.446 0.983				
Previous CVD	1.42 (0.71–2.84)	0.320				
Median GFR 2 PY^*^ (*n* = 231)	0.97 (0.96–0.99)	<0.001				
CKD	4.09 (2.04–8.20)	<0.001	2.13 (0.92–4.95)	0.078	3.05 (1.40–6.64)	0.005
Beta-blockers	1.98 (1.00–3.94)	0.050	1.52 (0.66–3.50)	0.329	1.48 (0.67–3.30)	0.336
ACE inhibitors	0.85 (0.35–2.05)	0.723				
ARB	1.53 (0.78–3.01)	0.218				
CCB	2.50 (1.20–5.22)	0.014	1.74 (0.76–3.98)	0.190	1.68 (0.76–3.70)	0.200
Thiazide diuretics	1.10 (0.47–2.55)	0.833				
Loop diuretics	2.54 (1.26–5.11)	0.009	1.17 (0.47–2.90)	0.740	1.22 (0.52–2.88)	0.648
MRA	1.68 (0.52–5.40)	0.382				
Antiplatelets	1.16 (0.59–2.28)	0.665				
Anticoagulants	1.57 (0.76–3.26)	0.225				
Lipid-lowering drugs	1.12 (0.57–2.20)	0.745				
Antidiabetics	0.82 (0.42–1.62)	0.572				

*Serum urate level at admission was not included due to the high rate of missing values (89/266, 33.5%). Ancestry was not included as no cases with gout were found in non-Caucasians*.

*Hyperuricemia was defined as median serum urate level ≥7 mg/dl in the previous 5 years. Chronic kidney disease was defined as median GFR <60 ml/min/1.73 m^2^ in the previous 2 years, estimated according to CKD-EPI formula*.

* *Quantitative variables*.

*OR, odds-ratio; 95% CI, 95% confidence interval; BMI, body mass index; CV, cardiovascular; PY, previous years; ACS, acute coronary syndrome; CHF, congestive heart failure; TIA, transient ischemic attack; PAD, peripheral artery disease; CVD, cardiovascular disease; GFR, glomerular filtration rate; CKD, chronic kidney disease; ACE, angiotensin-converting enzyme; ARB, angiotensin II receptor blockers; CCB, calcium channel blockers; MRA, mineralocorticoid-receptor antagonists*.

## Discussion

This is the first field study attempting to estimate the prevalence of gout in patients admitted for CV events (thus, at very high CV risk). Following chart reviews and face-to-face interviews using the ACR/EULAR clinical classification criteria (47), the disease rate in this population was 15%, present in one out seven of these patients. This data point is in contrast to the low rate of disease registration in patients' records and discharge reports. Hyperuricemia was the only predictor of gout in the admitted CV population; if the SU level was not available, then CKD would predict it. Gout was associated with older age, CKD, and use of diuretics. As for its characteristics, it was a long-standing disease but with few reported accumulative flares and low rates of tophi. Despite a CV background, the use of ULT and SU therapeutic levels was inadequate. In summary, the present study has unveiled a large population with gout and high CV risk but low standards—people who are candidates for dedicated education and management strategies.

In Western Europe and North America, the prevalence of gout in adults ranges between 0.3 and 4.8% (53). In Spain, a prevalence rate of 2.4% has been recently communicated (1). In patients with CKD, numbers are larger: 16.6% in an Irish cross-sectional study and 24.3% in a German cohort (54, 55). To date, there are scarce data on the prevalence of gout in a CV setting—a recent analysis of the Swedish heart failure registry reported 4.2% (56)—and no study had focused on patients admitted for CV events. In the present study, a prevalence rate of 15.0% has been obtained, six-fold the incidence in the Spanish adult population. Moreover, in the population hospitalized for CV events, gout is present in one out of seven patients, demonstrating the close relationship between CV and inflammatory diseases.

Compared to population-based or claims databases, a field study ensures high accuracy, here given the good performance that 2015 ACR/EULAR clinical criteria have shown for epidemiological studies in the absence of crystal-proven diagnosis (48). To reinforce this assertion, we audited discharge reports of CV events admissions for the inclusion of gout as a secondary diagnosis. The records at our hospital in the previous 2 years showed a limited number of cases registered, compared to the results obtained by interview in this field study. Numbers in discharge reports were similar to the prevalence of gout in general population. It is possible that the occurrence of a gouty flare during hospitalization influenced the inclusion. Other option would be the unawareness of gout as an independent CV risk factor.

Secondary CV prevention strategies are initiated when the first clinical event occurs. They aim to make a very high CV risk subside by reaching stricter lipid levels (residual lipid risk) and greater control of the atherothrombotic process (residual thrombotic risk). However, many patients still develop new events. The role of inflammation—measured by high-sensitivity C-reactive protein—as an independent risk factor (57) and the recently proven CV benefit of anti-inflammatory therapies such as blocking interleukin-1beta (58) have confirmed the existence of residual inflammatory risk (59). Gout is a well-established independent CV risk factor, but in one-third of the identified patients with gout and CV events, the diagnosis was not recorded (either in admission or in primary and specialized care records).

Gout is not included as a variable of interest in the different guidelines for CV management, which are firmly focused on traditional risk factors. Some of them include recommendations only to check SU levels, and in the case of heart failure following use of diuretics, the management of SU is guided (60, 61). Therefore, in line with the present results, identifying gout at the time of hospitalization is undoubtedly an excellent opportunity to start ULT and accordingly improve both secondary CV prevention and gout management itself. In this sense, our study shows hyperuricemia as the only independent predictor for the presence of gout in this population. Despite recommendations, which are relatively recent, 89 out of 266 patients (33.5%) did not have their blood tested for SU during admission, and 14 (5.3%) had not had it done in the previous 5 years either. If SU levels are not available, gout should be suspected if CKD is present, according to our results.

Gout was noted as long-standing but with a low number of flares and joints involved. In gout, flares are usually spaced at early stages, and as the crystal deposit grows, they become more frequent. Although a flare is usually intense enough to remember it, the number of subsequent episodes might not be retained in the same way, entailing a potential recall bias. Tophi were detected in 7.7% of patients, a rate considered low for a population with long-standing gout. This is probably an observer bias, as they were assessed by physical exam but not confirmed by imaging or sampling (62). Dedicated research to establish the prevalence of tophaceous gout in this population is needed, since tophi are a strong prognostic factor for mortality, mainly from CV origin (63).

Use of ULT and SU levels were inadequate in the sample. When admitted for CV events, patients initiate an intensive control of traditional CV risk factors. However, the poor management of gout observed is an added risk. Pagidipati et al. (64) found that after coronary revascularization, suffering from gout increased the risk of CV death by 19% and of all-cause death by 21%, despite adequate control of other risk factors. Appropriate gout management may likely help to reduce this risk (13, 15). Low-dose colchicine was also underused in the sample, despite its demonstrated anti-inflammatory properties (65) and potential clinical benefit as suggested by observational studies (44, 45).

Some strengths and limitations must be addressed. The sample size of this field study may be considered small. However, the minimal size was pre-calculated with respect to available prevalence estimates. Increasing numbers would add precision to the 95% CI estimation, though the current prediction of one gout patient in every five to ten CV inpatients is relevant and quite above the numbers in the general population (Spanish data, 2.4%). The value of a field study is to make disease diagnoses more correct, in contrast to medical records-based studies. Gout diagnosis by crystals was not feasible due to the epidemiological nature of the study, but the 2015 ACR/EULAR clinical criteria (47) have shown the best performance for epidemiological studies in the absence of crystal-proven diagnosis (48). Moreover, patients with known inflammatory arthritis were excluded to prevent misclassifications. Some gout cases could have been lost in cases of coexisting diseases (66); again, to differentiate them accurately, synovial fluid analysis would have been necessary. Determining the achievement of an SU target based on the time of admission may be problematic; in situations of acute inflammation, SU levels tend to decrease (67), whereas deterioration in renal function—occasionally occurring during a CV event—would cause an increase in SU levels. Also, study participants were not tested per protocol for SU levels, which were obtained from routine blood tests performed during hospitalization. In order to control this issue, the average of SU testing from the previous 5 years was obtained. The study centered on patients admitted for CV events, so the findings should only be generalized to the hospital setting. Some patients with established disease (TIA, stable angina, heart failure) may not require admission; as lower severity of atherosclerosis can be presumed, the prevalence of gout would then be lower. However, it is convenient to replicate the results in the outpatient setting.

## Conclusions

Gout was present in 15% of hospitalized patients for CV events, a prevalence six times higher than in the Spanish adult population and hospital records. A significant rate of under-registration was detected, both in patients' records and discharge reports. Hyperuricemia was the only predictor of gout in admitted CV population; if the SU level is not available, CKD would be taken as a predictor. Gout was long-standing but with a low number of flares and involved joints, and suboptimal management was identified. Therefore, this field study was able to uncover a subpopulation with high CV risk, candidates for both secondary CV prevention strategies and dedicated gout management.

## Data Availability Statement

The raw data supporting the conclusions of this article will be made available by the authors, without undue reservation.

## Ethics Statement

The studies involving human participants were reviewed and approved by Alicante Institute of Sanitary and Biomedical Research (ISABIAL) (2018/07 act). The patients/participants provided their written informed consent to participate in this study.

## Author Contributions

IC, MG-G, and MA designed the study project and analyzed and interpreted the results. IC and MG-G acquired the data. MG-G and MA wrote the first draft of the manuscript. All authors contributed, revised, and approved the final version. All authors contributed to the article and approved the submitted version.

## Conflict of Interest

MA declares consultancies and speaking fees from Menarini, Grünenthal, and Horizon. The remaining authors declare that the research was conducted in the absence of any commercial or financial relationships that could be construed as a potential conflict of interest.
