# Supplementary material for: Gout Is Prevalent but Under-Registered Among Patients With Cardiovascular Events: A Field Study
Source: Front Med (Lausanne). 2020 Sep 29;7:560. doi: 10.3389/fmed.2020.00560 (PMC7552997; doi:10.3389/fmed.2020.00560)
Supplement: Supplementary file 1 [file Data_Sheet_1.docx]

**Gout is prevalent but under-registered among patients with cardiovascular events: a field study**

**SUPPLEMENTARY APPENDIX**

**FULL LIST OF VARIABLES**

**Primary outcome variables:**

- Presence of gout:
  - Face-to-face interview, following 2015 ACR/EULAR clinical classification criteria.
  - Patients’ records.
  - Discharge reports, including M10.X codes according to International Classification of Diseases 10^th^ edition.

**Secondary outcome variables:**

- Serum urate (SU) level at the time of the cardiovascular (CV) event (mg/dl).
- Median SU level in the previous five years (mg/dl).
- Years since the first gout flare.
- Number of flares.
- Presence of tophi (at the time of admission).
- Use of urate-lowering therapy (current or previous, dose and type: allopurinol, febuxostat, benzbromarone).
- Use of colchicine (current or previous) and use of NSAIDs to treat the gout flare.

**Additional explanatory variables:**

- CV event and date of occurrence:
  - (i) acute coronary syndrome or coronary artery disease that requires revascularisation.
  - (ii) new or congestive heart failure.
  - (iii) stroke or transient ischemic attack.
  - (iv) peripheral artery disease, acute or chronic that requires revascularisation.
- Previous CV events (in patients’ record) and date of occurrence.
- Glomerular filtration rate (GFR) at the time of the CV event (ml/min/1.73 m2, estimated according to CKD-EPI formula).
- Median GFR in the previous two years (ml/min/1.73 m2, estimated according to CKD-EPI formula).
- Presence of chronic kidney disease (CKD) defined as the decrease in the kidney function expressed as GFR <60 ml/min/1.73m2.
- Age (years).
- Gender.
- Height (m).
- Weight (kg).
- Body mass index (kg/m2).
- Ancestry.
- CV risk factors as hypertension, dyslipidemia or diabetes mellitus (by clinical diagnosis in patients’ record or taking specific treatment).
- Current use of tobacco.
- Current use of alcohol.
- Current use of drugs (beta blockers, angiotensin converting enzyme inhibitors, angiotensin II receptor blockers, calcium channel blockers, thiazides, loop diuretics, aldosterone antagonists, antiplatelets, anticoagulants, lipid-lowering drugs, antidiabetic drugs).
